# Evaluation of droplet digital PCR for characterizing plasmid reference material used for quantifying ammonia oxidizers and denitrifiers

**DOI:** 10.1007/s00216-013-7546-1

**Published:** 2014-02-04

**Authors:** Lianhua Dong, Ying Meng, Jing Wang, Yingying Liu

**Affiliations:** 1 National Institute of Metrology, Beijing, 100013 China; 2 Hubei Institute of Measurement and Testing Technology, 430223 Wuhan, China

**Keywords:** Plasmid DNA reference material, Droplet digital PCR, Isotope dilution mass spectrometry, Ammonia oxidizer, Denitrifier

## Abstract

DNA reference materials of certified value have a critical function in many analytical processes of DNA measurement. Quantification of *amoA* genes in ammonia oxidizing bacteria (AOB) and archaea (AOA), and of *nirS* and *nosZ* genes in the denitrifiers is very important for determining their distribution and abundance in the natural environment. A plasmid reference material containing *nirS*, *nosZ*, *amoA*-AOB, and *amoA*-AOA is developed to provide a DNA standard with copy number concentration for ensuring comparability and reliability of quantification of these genes. Droplet digital PCR (ddPCR) was evaluated for characterization of the plasmid reference material. The result revealed that restriction endonuclease digestion of plasmids can improve amplification efficiency and minimize the measurement bias of ddPCR. Compared with the conformation of the plasmid, the size of the DNA fragment containing the target sequence and the location of the restriction site relative to the target sequence are not significant factors affecting plasmid quantification by ddPCR. Liquid chromatography–isotope dilution mass spectrometry (LC–IDMS) was used to provide independent data for quantifying the plasmid reference material. The copy number concentration of the digested plasmid determined by ddPCR agreed well with that determined by LC–IDMS, improving both the accuracy and reliability of the plasmid reference material. The reference value, with its expanded uncertainty (*k* = 2), of the plasmid reference material was determined to be (5.19 ± 0.41) × 10^9^ copies μL^−1^ by averaging the results of two independent measurements. Consideration of the factors revealed in this study can improve the reliability and accuracy of ddPCR; thus, this method has the potential to accurately quantify DNA reference materials.

**Electronic supplementary material:**

The online version of this article (doi:10.1007/s00216-013-7546-1) contains supplementary material, which is available to authorized users.

## Introduction

DNA reference materials of certified value have a critical function in many analytical processes involving nucleic acid analysis. Many important challenges have contributed to the need for accurate and reliable DNA reference materials, for instance legislative requirements for analysis of genetically modified organisms (GMOs). qPCR techniques have been used for quantification of GMOs in several studies [1, 2]. However, qPCR is unable to quantify the GM content, usually the ratio of the transgenic gene copy number to the endogenous gene copy number, without a DNA reference standard containing the target gene. The JRC’s Institute of Reference Material Measurement (IRMM) and the American Oil Chemists’ Society (AOCS) have developed more than 40 different kinds of GM reference material. Other examples of applications requiring DNA reference material include clinical applications, scene-of crime genotyping, and microbial contamination of food. All of these tests could benefit greatly from more accurate and reliable DNA reference material.

In environmental microbiology, many studies focus on the distribution and abundance of specific functional microbial groups involved in nitrification and denitrification [3–5]. Nitrification and denitrification are essential, microbe-driven processes in the global nitrogen cycle. Ammonia oxidization, the first and rate-limiting step of nitrification, is performed by ammonia oxidizing bacteria (AOB) and ammonia oxidizing archaea (AOA) [6]. For both the AOA and AOB, the *amoA* gene encoding the a-subunit of ammonia monooxygenase has been widely used as a functional marker for investigating their distribution and abundance in natural environments [6, 7]. Denitrification is the enzymatic, stepwise reduction of nitrate and nitrite to the gases nitric oxide (NO), nitrous oxide (N_2_O), and nitrogen gas (N_2_). Nitrous oxide (N_2_O) and nitric oxide (NO) are intermediate products in the denitrification pathway. N_2_O, with a global warming potential approximately 296 times higher than that of carbon dioxide, is an important greenhouse gas [8]. The *nosZ* gene, encoding the catalytic subunit of N_2_O reductase, and the *nirS/K* gene, encoding nitrite reductase, are widely used as functional markers for studying the abundance of bacteria able to metabolize N_2_O and NO, to better understand the main causes of N_2_O and NO emissions [9–11].

Real-time quantitative PCR (qPCR) is the most popular approach used to target the above functional genes to quantify the abundance of the functional groups [9, 10, 12]. However, an external standard or a calibrant is needed to quantify the environmental sample when using qPCR. In the absence of a certified DNA reference material, one usually develops one’s own plasmid standard [12, 13] or extracts genomic DNA from pure culture [9], and quantifies the standard DNA concentration by measuring absorbance at 260 nm by use of a UV spectrophotometer. UV spectrophotometry is an established method for measuring nucleic acids; however, common contaminants of DNA extracts, for example proteins, RNA, and salts, can increase absorbance at 260 nm, resulting in overestimation of DNA concentration. Moreover, UV spectrophotometers cannot distinguish between single-stranded DNA and double-stranded DNA in solution, nor between target DNA and other, potentially contaminating sources of DNA or RNA. Thus, this method has limitations that may contribute to inaccuracy of DNA concentration estimates, and use of such a DNA standard to quantify the abundance of functional microbial groups in the environmental sample will result in inaccurate information. This will lead to incomparability of results from different research groups.

There is a need for an accurate and traceable method that can be used for characterization of DNA certified reference material, for effective comparison of quantitative measurements, quality control in laboratory routine analysis, and method validation. Digital PCR (dPCR) is a relatively new technology, requiring no external calibrators, for measuring the absolute and relative copy numbers of target DNA [14, 15]. Digital PCR (dPCR) transforms the exponential, analogue signal of classic PCR into a linear, digital signal, retaining the single-molecule sensitivity of PCR. Single molecules are isolated by dilution and individually amplified by PCR; each product is then analyzed separately. This is achieved by partitioning a sample before PCR amplification. The distribution of target DNA molecules among the partitions follows Poisson statistics, and at the so-called limiting dilution most reactions contain either one or zero target DNA molecules [16–18]. An absolute target sequence quantity can be estimated [16, 17]. Droplet digital PCR (ddPCR) is a droplet-based form of dPCR that has recently been commercialized. Pinheiro [19] reported it has high accuracy and precision for quantifying genomic DNA concentration. However, little information is available regarding use of ddPCR for quantifying plasmid DNA.

Another accurate and traceable nucleic acid quantification approach is liquid chromatography–isotope dilution mass spectrometry (LC–IDMS) [20, 21]. This method overcomes the lack of DNA standards in suitable quantities by using deoxynucleotide monophosphates (dNMPs) and isotopically-labeled dNMPs (LdNMPs) as the calibrants and internal standards, respectively. Additionally, this approach provides full traceability to the International System of units (SI). IDMS has been well established elsewhere as providing highly reliable quantitative trace analysis [22, 23]; thus, it has become the method of choice for the quantification of analytes in primary standards for many national measurement institutes.

In this study, we constructed a plasmid containing the *amoA* gene of AOA (A-*amoA*) and AOB (B-*amoA*), and the *nosZ* and *nirS* genes of denitrifiers. Droplet digital PCR (ddPCR) was evaluated for characterization of the plasmid reference material. The dynamic range and factors involved in ddPCR measurement accuracy and bias were investigated. Additionally, LC–IDMS was used to provide independent data for comparison of the ddPCR result. The results reveal the DNA copy number concentration measured by ddPCR is comparable with LC–IDMS for quantifying the mass concentration.

## Material and methods

### Soil characteristics and sampling

Soil was collected from the field in cotton–spring maize (*Triticum aestivum L.*) rotation at the Chinese academy of agriculture science, PingGu, Beijing, China in May 2012, before the cotton planting. The soils were coarse loam. The soil pH was 6.1, as determined by a 1:1 soil:water suspension. The total organic C concentration was 1.02 g kg^−1^, and the total N concentration was 0.99 g kg^−1^, as determined by combustion (Leco CNS-1000). Soil was frozen at −20 °C to limit biological activity. At the start of the experiment, soil was thawed at room temperature, homogenized, and passed through a 2-mm sieve.

### DNA extraction and purification

Total soil genomic DNA was extracted and purified by using a MOBIO DNA extraction kit (MOBIO Laboratories Inc., Carlsbad, CA) according to the manufacturer’s instructions. 0.25 g soil mixed with solution C1 was vortexed in the Power Bead tube at maximum speed for 10 min, then centrifuged at 10,000 *g* for 30 s at room temperature. The supernatant was transferred to a clean 2 mL collection tube and mixed with 250 μL solution C2. The mixture was then centrifuged at 10,000 *g* for 1 min, and 600 μL of the supernatant was transferred to a new 2 mL collection tube. 200 μL solution C3 was added to the tube, which was then incubated at 4 °C for 5 min, and then centrifuged at room temperature for 1 min at 10,000 *g*. 750 μL of the supernatant was pipetted into a clean 2 mL collection tube and mixed with 1200 μL solution C4. Approximately 675 μL was loaded into a spin filter (provided in the kit) and centrifuged at 10,000 *g* for 1 min. The flow-through was discarded, an additional 675 μL supernatant was added to the spin filter, and centrifugation was repeated. 500 μL solution C5 was added to the filter to wash the DNA, and the mixture was then centrifuged for 30 s at 10,000 *g*. The flow-through was discarded, and 100 μL DNA-free PCR-grade water was loaded into the center of the filter membrane to dissolve the DNA. The spin filter was placed in a clean 2 mL collection tube and centrifuged at room temperature for 30 s at 10,000 *g*. Finally, the dissolved DNA was stored at −20 °C, ready for any downstream application.

### PCR amplification of target genes from soil DNA and plasmid construction

A plasmid containing A-*amoA*, B-*amoA*, *nosZ* and *nirS* gene fragments was cloned by use of overlapping PCR [24]. The primer pairs BA-F/B-R (696 bp of B-*amoA* gene), A-F/BA-R (689 bp of A-*amoA* gene), ZS-F/Z-R (313 bp of *nosZ* gene), and S-F/ZS-R (455 bp of *nirS* gene) used to clone the four fragments were designed based on the sequences of the B-*amoA*, A-*amoA*, *nosZ*, and *nirS* genes, respectively. The PCR amplicons were obtained using three rounds of PCR. In the first round of PCR, the amplicons of B-*amoA*, A-*amoA*, *nosZ*, and *nirS* genes were amplified with primers BA-F/B-R, A-F/BA-R, ZS-F/Z-R, and S-F/ZS-R (Table 1), respectively. The amplicons of the four gene fragments were then purified using the Gel Extraction Purification Kit (Tangen Biotech, Beijing, China). In the second round of PCR, amplicons of the A-*amoA* and B-*amoA* genes were connected using primers BA-F/BA-R, with the first round of purified PCR products (A-*amoA* and B-*amoA*) as templates. Meanwhile, amplicons of the *nosZ* and *nirS* genes were connected using primers ZS-F/ZS-R, with the first-round PCR amplicons (*nosZ* and *nirS*) as templates. The two PCR amplicons of B-*amoA*–A-*amoA* and *nosZ*–*nirS* were purified using the same purification kit. In the third round of PCR, amplicons of B-*amoA*–A-*amoA* and *nosZ*–*nirS* were connected using primers BA-F/ZS-R, with the second-round purified PCR amplicons (B-*amoA*–A-*amoA* and *nosZ*–*nirS*) as templates.

**Table 1:** Primer sequence for amplification of A-amoA, B-amoA, nosZ and nirS gene from soil and construction of the plasmid standard

Primers	Target	Sequence (5ʹ to 3ʹ)	Size of PCR amplicon (bp)
BA-F	*amoA* (AOB)	GGHGACTGGGAYTTCTGG	696
B-R		CGTCTAAGCCAGACCATTAStctagaCCTCKGSAAAGCCTTCTTC	
A-F	*amoA* (AOA)	GAAGAAGGCTTTSCMGAGGtctagaSTAATGGTCTGGCTTAGACG	689
BA-R		CTGGCTGTCGAKGAACARSGWgcggccgcGCGGCCATCCATCTGTATGT	
ZS-F	*nosZ*	ACATACAGATGGATGGCCGCgcggccgcWCSYTGTTCMTCGACAGCCAG	313
Z-R		CCSGTYTCCTTSACGTTSACggatccATGTCGATCARCTGVKCRTTYTC	
S-F	*nirS*	GARAAYGMBCAGYTGATCGACATggatccGTSAACGTSAAGGARACSGG	455
ZS-R		GASTTCGGRTGSGTCTTGA	

The 50 μL reaction mixture was comprised of 2 × 25 μL Taqman Environmental Master Mix 2.0 (Life Technologies), 1 μL 20 μmol L^−1^ forward and reverse primers, and 5 ng template DNA. The first and third round of PCR thermal cycling consisted of a 5 min activation period at 95 °C, followed by six cycles of a touch-down PCR thermal profile of 45 s at 95 °C for denaturation, 60 s at 53–58 °C for annealing, and 60 s at 72 °C extension; then 30 cycles of 45 s at 95 °C for denaturation, 60 s at 53 °C for annealing, and 2 min at 72 °C extension; and a final step of 10 min at 72 °C. The second-round PCR program consisted of a 5 min activation period at 95 °C, followed by 30 cycles of a PCR thermal profile of 45 s at 95 °C for denaturation and 60 s at 55 °C for annealing, and 2 min at 72 °C extension, and a final step of 10 min at 72 °C. All PCR amplifications were performed on a PTC-200 thermocycler (BioRad, CA).

After the third round of PCR, the integrated PCR amplicon was purified using the Gel Extraction Purification Kit (Tangen Biotech, Beijing, China), and ligated into pEASY-T3 vector and transformed to *E. coli*. Sequencing analysis of the cloned DNA was performed using the ABI 3730 XL Genetic Analyzer (Applied-Biosystems) by BGI Co., Ltd. (Beijing, China). Endogenous restriction enzymes *BamHI* and *XbaI* were used to digest the plasmid (pNIM-003), to check the correction of the fragment size.

### Droplet digital PCR (ddPCR)

Quantification of the plasmid pNIM-003, using ddPCR targeting the AOB gene, was performed on QX100 (BioRad), with the primer and probe (A189F/amoA-2R' and A337) listed in Table 2. The ddPCR workflow and data analysis were performed as described by Pinheiro [19]. Amplification conditions consisted of a 10 min activation period at 95 °C, followed by 40 cycles of a three-step thermal profile of 15 s at 94 °C for denaturation, 60 s at 55 °C for annealing, and 60 s at 72 °C extension, and a final 10 min inactivation step at 98 °C. After thermal cycling, plates were transferred to a droplet reader (Bio-Rad) to read the droplets.

**Table 2:** Primer and probe sequence and concentration for quantitative PCR and droplet digital PCR

Primer or probe	Target	Sequence (5ʹ to 3ʹ)	Concentration (nmol L^−1^)	Product size (bp)	Ref.
A189F	*amoA* (AOB)	GGGGTTTCTACTGGTGGT	200	670	(29)
amoA-2R'		CCCCTCKGSAAAGCCTTCTTC	200		
A337		FAM-CCCCTCKGSAAAGCCTTCTTC-TAMRA	200		
Arch-amoAF	*amoA* (AOA)	STAATGGTCTGGCTTAGACG	200	635	(10)
Arch-amoAR		GCGGCCATCCATCTGTATGT	200		
nosZ1F	*nosZ*	WCSYTGTTCMTCGACAGCCAG	200	259	(17)
nosZ1R		ATGTCGATCARCTGVKCRTTYTC	400		
cd3aF	*nirS*	GTSAACGTSAAGGARACSGG	400	426	(36)
ZS-R		GASTTCGGRTGSGTCTTGA	200		

The final copy number of the plasmid determined by ddPCR was calculated by use of Eq. (1).

1 $$ T=\frac{-D}{V_P}\times 1\mathrm{n}\left(1-\frac{P}{N}\right)\kern0.5em \mathrm{or}\kern0.5em \left(T=\frac{D\times M}{V_P}\right) $$

Where *T* is the copy number per microliter, *D* is the dilution factor combining both the factor used to dilute the DNA during PCR preparation and the factor used to further dilute the DNA with the PCR master mixture, *V*_*p*_ is the droplet volume, *P* is the number of positive droplets, *N* is the total number of accepted droplets, and *M* is the copy number per droplet. The uncertainty for *T*, related to the volume of the droplet, copy number per droplet, and the dilution factor, was calculated by use of Eq. (2). The relative standard uncertainty for *M* was estimated by Eq. (3) [19]. The relative standard uncertainty of the droplet volume was determined from analysis of an individual droplet volume measured using a Zeiss Observer Z1 microscope (please refer the Electronic Supplementary Material Fig. S4), and the calibration of that microscope. The digital image was analyzed using ImageJ v1.34 s, and the process used for determining the equivalent circular diameter and the equivalent spherical volume of a sphere using ImageJ was as described in a previous report [19].

2 $$ \frac{u_T}{T}=\sqrt{{\left(\frac{u_D}{D}\right)}^2+{\left(\frac{u_M}{M}\right)}^2+{\left(\frac{u_{V_P}}{V_P}\right)}^2} $$

3 $$ \frac{u_M}{\overline{M}}=\frac{ SD}{\overline{M}\sqrt{n}} $$

### Enzymatic restriction of plasmid DNA

Three restriction enzymes were chosen to investigate the effect of the conformation and the size of the fragment on quantification using ddPCR. The restriction enzymatic sites are labeled in Fig. 2. *BamH*1 was used to linearize the plasmid, and *EcoR*1 to fragment the DNA target into small pieces. The combination of *EcoR*1 and *Xba*1 was used to cut the target fragment at equal distances from the forward and reverse primer. Enzymatic digestion of the mixture with either *BamH*1, *ECOR*1, or *ECOR*1 with *Xba*1 (Takara, China) was performed as follows: 10 × 2 μL buffer, 1 μL restriction enzyme, 10 μL plasmid DNA, and 7 μL ddH_2_O. No template control (NTC) was prepared by adding 10 μL TE_0.1_ (10 mmol L^−1^ Tris–HCl, 0.1 mmol L^−1^ EDTA) instead of the DNA solution, and no enzyme control (NEC) was made by pipetting 1 μL TE_0.1_ in place of the enzyme when preparing the enzymatic master mixture. The enzymatic time was 1 h. After the enzymatic reaction, the DNA was diluted to the appropriate concentration for the ddPCR analysis procedure described above.

For the dynamic range of ddPCR, the plasmid digested with *EcoR*1 was diluted to 2,177,456, 215,982, 22,315, 2078, 235, 22.7, or 2.3 copies 20 μL^−1^ ddPCR (from dilution 1 to dilution 7, labeled S1–S7). Dilutions were ascertained using UV measurement. Seven dilutions were made of the enzyme-digested plasmid, and each dilution had four replicates; thus, a total of 30 reactions plus two NTCs were prepared to evaluate the dynamic range. Meanwhile, undigested pNIM-003 was diluted to dilutions 1–6 (labeled as S1–S6) for use as the template in the NEC.

### Preparation of standards and isotopically-labeled internal standards for LC–IDMS

The dNMP standards (approximately 10 mg) were weighed on a six-figure calibrated analytical balance and dissolved in 10 g water. Detailed information about the purity and certificate of the dNMP standard was described in our previously published paper [21]. A mixed stock standard solution containing the desired concentrations of each dNMP was prepared gravimetrically. The concentration of dNMP in the stock standard was corrected for purity. A mixed stock standard containing 4.5 μg g^−1^ dAMP, 6.3 μg g^−1^ dCMP, 8.0 μg g^−1^ dGMP, and 5.4 μg g^−1^ dTMP was prepared. These concentrations correspond to the amount of dNMP in the plasmid DNA solution (assuming total digestion of the plasmid DNA to its constituent dNMPs). The preparation of the LdNMPs was nearly the same as that for the natural dNMPs. A final mixed stock solution of LdNMPs containing 7.0 μg g^−1^ dAMP, 14.0 μg g^−1^ dCMP, 14.3 μg g^−1^ dGMP, and 9.9 μg g^−1^ dTMP was prepared. The mixed solution was used to prepare the calibration and sample blends for the IDMS experiments. The isotopic purity of the LdNMPs was stated to be higher than 98 % by the certificate provided by the manufacturer.

Sample (diluted plasmid DNA) and calibration blend solutions were prepared by gravimetrically adding equal amounts of the LdNMP standard to the plasmid DNA sample and the mixed dNMP standard. Briefly, 50 μL LdNMP solution and 50 μL dNMP standard were weighed and mixed as the calibration blend; 50 μL plasmid DNA sample and 50 μL LdNMP solution were weighed and mixed to form the sample blend.

### Ultrasonic treatment and digestion of the target sample

For the ultrasonic treatment, 100 μL sample blend or calibration blend was sheared by a Covaris S2 system (Covaris, Applied Biosystems, Carlsbad, CA, USA), in a 1.5-mL tube on the 1.5 tray (Covaris, Applied Biosystems). The conditions of the ultrasonic treatment for plasmid were: intensity, 5; treatment time, 25 min [21]. The duty cycle and the number of cycles per burst were 10 % and 200, respectively.

The digestion master mixture consisted of 5 μL phosphodiesterase I (0.02 U μL^−1^) and 5 μL digestion buffer containing 300 mmol L^−1^ Mg(Ac)_2_, 10 mmol L^−1^ ammonium acetate, and 100 mmol L^−1^ Tris–HCl. The sample or calibration blend (50 μL) was added to this master mixture, and the final mixture was incubated at 37 °C for 1 h. This step was performed in triplicate for both the sample and the calibration blends. Blanks were prepared by adding ultraclean water in place of the sample or calibration blends when preparing the master mixture.

### Liquid chromatography–mass spectrometry (LC–MS)

The LC–MS system consisted of an ultra-high-performance liquid chromatography (UPLC) Agilent 1290 quaternary pumping module, with an integral autosampler, a column oven, and an AB5500 triple quadrupole tandem mass spectrometry detector (AB SCIEX). Separation of the four dNMPs was achieved using an ACQUITY UPLC HSS T3 C18, 1.8 μm, 2.1 mm × 150 mm analytical column (Waters). The mobile phase (solvent A) consisted of 0.01 mol L^−1^ ammonium acetate, buffered to pH 3.5 with acetic acid and pumped at a flow of 0.2 mL min^−1^. The organic phase (solvent A) was acetonitrile. The mobile phase gradient for separation of the four dNMPs was: 0–4 min, 3 % solvent A, 97 % solvent B; 9 min, 15 % solvent A, 85 % solvent B; 12 min, 15 % solvent A, 85 % solvent B; 13 min, 3 % solvent A, 97 % solvent B; 15 min, 3 % solvent A, 97 % solvent B. Sample aliquots of 2 μL were injected. The MS–MS conditions and the MS data acquisition modes used in this study are shown in Table 3. The dNMPs and LdNMPs were introduced into the mass spectrometer via the LC system. For the MS–MS analysis of the dNMPs, the instrument was operated in multiple-reaction-monitoring mode (MRM), whereby the precursor to product ion was same as described in a previous report [21].

**Table 3:** Parameters used for the mass spectrometry analysis

Ionization mode	Electrospray, positive ion mode
Ion spray voltage (V)	5500
Curtain gas (L h^−1^)	30
Collision gas	Medium
Temperature (°C)	650
Ion source gas 1 (L h^−1^)	50
Ion source gas 2 (L h^−1^)	40
Declustering potential (V)	60
Entrance potential (V)	7
Collision energy (V)	14
Collision cell exit potential (V)	18

### Measurement equation for the plasmid DNA mass fraction

The calculation for the final mass fraction of each dNMP in the digested-plasmid DNA solution was as described in a previous paper [20]. The mass fraction of the target plasmid DNA was calculated by converting the mass fraction of each dNMP according to its mole fraction in the plasmid, using Eq. (4).

4 $$ {w}_{X, DNA}=\frac{w_{X, dNMP}}{M_{R, dNMP}}\times \frac{M_{R, DNA}}{N_{dNMP}} $$

Where *w*_*X,dNMP*_ is the mass fraction of dNMP in the hydrolysate (ng mg^−1^), *w*_*X,DNA*_ is the mass fraction of double-stranded DNA (ng mg^−1^), *M*_*R,DNA*_ is the molecular weight of the plasmid pNIM-003 (3,313,860 g mol^−1^), *N*_*dNMP*_ is the number of molecules in the specific nucleotide in the pNIM-003 plasmid, and *M*_*R,dNMP*_ is the average molecular weight (hydrated) of the specific dNMP (g mol^−1^).

### Homogeneity and stability study for the plasmid reference material

The homogeneity study of the reference value was performed under repeatability conditions, using 15 bottles randomly taken from the entire batch and tested in random order. Three subsamples were taken from each bottle and each was analyzed in triplicate. The measurements were performed by ddPCR, using the AOB PCR assay, and the minimum sample intake for ddPCR is 4 μL. ANOVA was used to assess the between-bottle standard deviation (*u*_*bb*_). Further information regarding the homogeneity test is in the Electronic Supplemental Material (Section 1, Homogeneity study).

The long-term stability of plasmid pNIM-003 during storage was monitored at the National Institute of Metrology, China, for one year, using ddPCR with AOB assay. The plasmid concentration was measured after 0, 1, 2, 4, 7, and 12 months storage at −70 °C, and the data was used to assess the stability over long storage times and to estimate the uncertainty contribution of the stability according to the ISO guide 35 [25]. For detailed information please see the Electronic Supplemental Material (Section 2, Stability study).

## Results and discussion

### Plasmid construction and verification

**Fig. 1:**
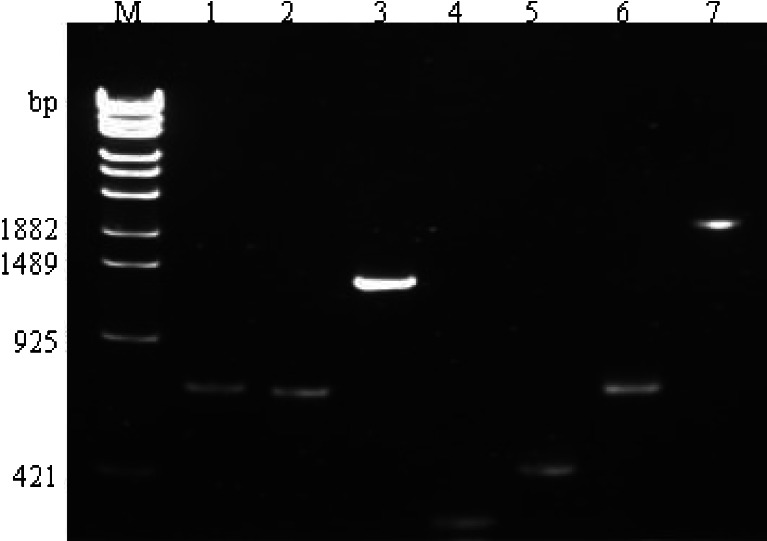
Electrophoresis for PCR products of *amoA* (AOB), *amoA* (AOA), *nosZ* and *nirS*

Four target genes of B-*amoA*, A-*amo*A, *nirS* and *nosZ*, with the expected sizes of 696 bp, 689 bp, 313 bp and 455 bp, respectively (lane 1, 2, 4 and 5 in Fig. 1), were successfully amplified from soil. The overlapping PCR (SOE PCR) amplicons of A-*amo*A–B-*amoA* and *nirS*–*nosZ*, with expected sizes of 1340 bp and 719 bp, are shown in lane 3 and lane 6, respectively. Lane 7 shows the final SOE PCR product of A-*amo*A–B-*amoA*–*nirS*–*nosZ*, with the correct size of 2010 bp. The electrophoresis results of these PCR amplicons indicate that no other unspecific amplification occurred.

**Fig. 2:**
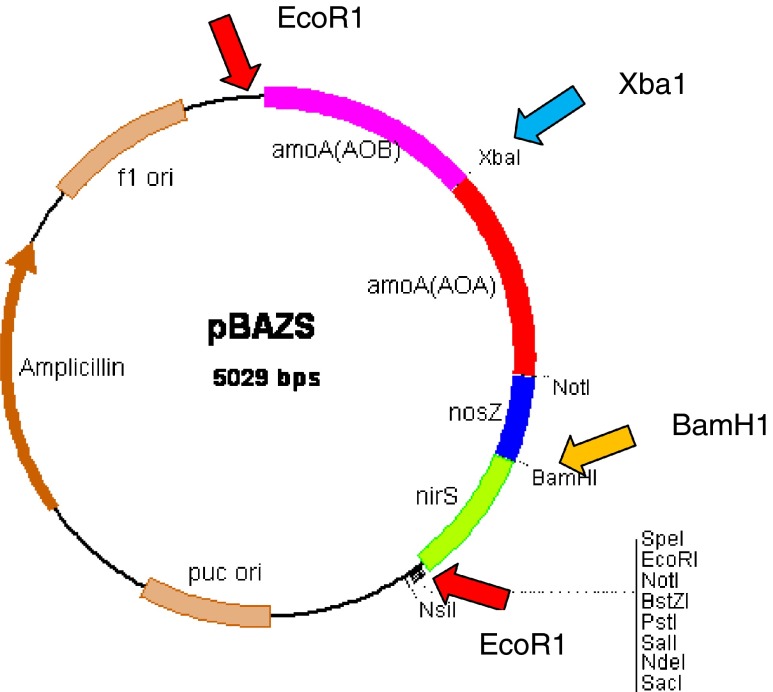
Construction map of the plasmid pNIM-003

**Fig. 3:**
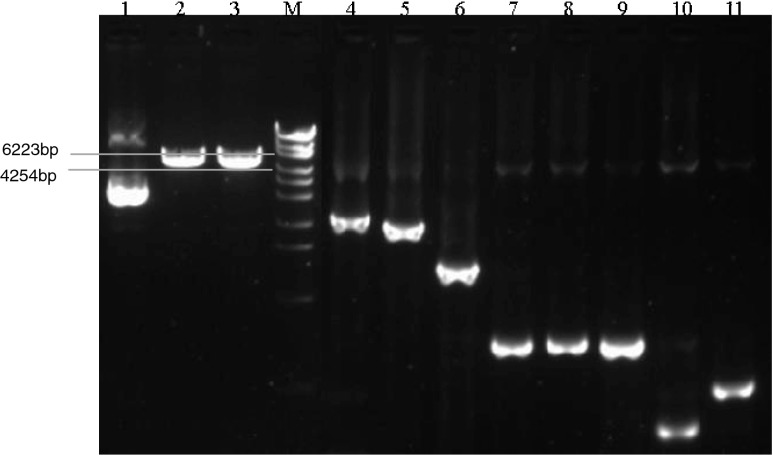
Identification of recombinant plasmid by restriction digestion and PCR

The construction map shown in Fig. 2 was generated according to the sequence of pNIM-003, which indicates that the four target genes of B*-amoA,* A*-amoA, nirS*, and *nosZ* were ligated from 5ʹ to 3ʹ with a single copy. The total size of the recombinant plasmid is 5029 bp. The plasmid purified from *E. coli* was mainly supercoil plasmid, with a small proportion of open circular plasmid (lane 1 in Fig. 3). The size of the linear plasmid digested by *BamH*1 or *Xba*1 (lanes 2 and 3 in Fig. 3) was between 6223 bp and 4254 bp, as expected.

PCR confirmation is shown in Fig. 3. Four single-gene fragments (B*-amoA,* A*-amoA, nirS* and *nosZ*, in lanes 8, 9, 10, and 11, respectively), two connected-gene fragments (B*-amoA*–A*-amoA* in lane 6, *nirS*–*nosZ* in lane 7)*,* and the four-connected-gene fragment (B*-amoA*–A*-amoA*–*nirS*–*nosZ* in lane 5) were successfully amplified using the plasmid as the template. After confirmation by restriction digestion, PCR, and sequencing, the purified plasmid was ready to be characterized, with a reference value of copy number μL^−1^.

### Homogeneity and stability of the plasmid reference material

The result of the homogeneity testing is summarized in Table S1 (Electronic Supplementary Material). First, normal probability plots and histograms were used to establish that the data followed a normal distribution. The individual data and the bottle means from the homogeneity study measured for pNIM-003 were normally distributed. No outliers were detected for these data using the Grubbs tests (95 % confidence level). The ANOVA test indicated that the plasmid reference material is homogeneous and the relative uncertainty of the homogeneity (*u*_*bb,rel*_) is 0.15 %.

The result indicating a year’s long-term stability of the pNIM-003 is shown in Fig. S1 and Table S2. According to the principle of CRM for stability assessment, described in ISO guide 35, the plasmid RM is stable under the storage conditions. The relative standard uncertainty of the long-term stability (*u*_*s,rel*_) was approximately 3 % for a shelf life of one year, and was used as the contribution to the uncertainty budget from the instability of CRMs during storage.

### Enzymatic restriction effect on plasmid quantification by ddPCR

**Fig. 4:**
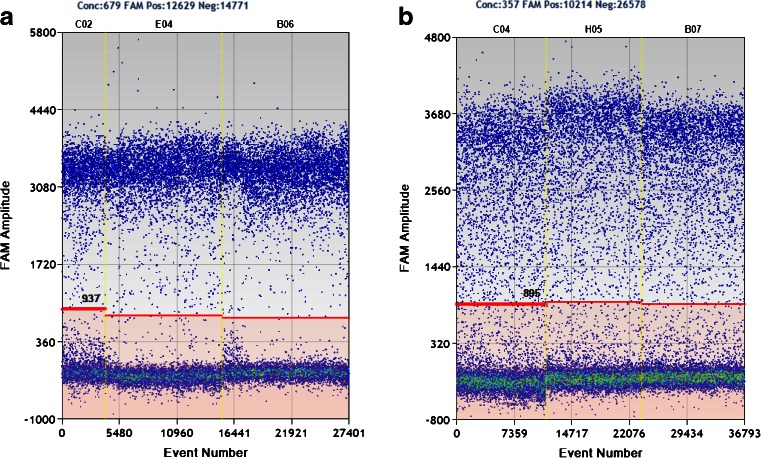
Effect of restriction digestion on plasmid quantification by ddPCR

One-dimensional scatter plots for selected wells with digested or undigested plasmid are listed in Fig. 4. Ideally, during the reaction, the rain dots with a high fluorescence signal, representing the positive droplets, aggregate and separate clearly from the negative droplets with a background fluorescence signal. However, there is a smear between the negative and positive droplets in undigested treatment (NECs, Fig. 4a), indicating an amplification delay for these droplets. This will lead to underestimation of the true copy number.

It has been reported that amplification efficiency can be improved by digestion of target genomic DNA [26] and plasmid DNA [14] with restriction endonuclease enzymes. In this study, we found it was necessary to digest the plasmid DNA when using ddPCR: not only was the amplification efficiency greatly improved by enzyme digestion of the plasmid (Fig. 4), but the concentration of digested plasmid determined by ddPCR was significantly different from the determined concentration of undigested plasmid (Table 4). This suggests enzymatic digestion of plasmid can affect ddPCR quantification. We deduced that it is relatively easy for the primer or probe and the DNA polymerase to bind to the target region of the linear plasmid compared with that of the undigested supercoil plasmid, because of the exposure of the target sequence of the digested plasmid. Thus, the number of delayed amplification droplets greatly decreased, and the positive droplets increased, resulting in an increase in the measured copy number concentration. The copy number of digested linearized plasmid is significantly higher than that of undigested non-linearized plasmid (Table 4); therefore, the conformation of the plasmid is an important factor for accurate quantification by ddPCR.

**Table 4:** Measured copy number concentration of digested and undigested plasmid obtained using ddPCR with three different restriction endonuclease enzyme digestions

	Digested plasmid copy number concentration (copies μL^−1^)^a^	Undigested plasmid copy number concentration (copies μL^−1^)^a^
BamH1	(4.69 ± 0.17) × 10^9^*	(2.05 ± 0.08) × 10^9^†
EcoR1	(4.77 ± 0.16) × 10^9^*	(2.12 ± 0.06) × 10^9^†
EcoR1 + XbaI	(4.70 ± 0.16) × 10^9^*	(2.31 ± 0.05) × 10^9^†

^a^Mean and standard deviation from five replicates

*,†The same symbol indicates no significant difference determined by *t*-test

In addition to plasmid conformation, we also considered other factors that may affect plasmid quantification by ddPCR, including the location of the restriction site relative to the target sequence and the size of DNA fragment containing the target sequence. However, there was no significant difference in the one-dimensional scatter plots for different restriction enzyme digestion (Fig. 4a). Furthermore, the T-test showed there was no significant difference in the plasmid copy number concentrations determined by ddPCR for the three different restriction enzyme treatments (Table 4). Therefore, to obtain accurate plasmid copy number concentrations using ddPCR, linearizing the plasmid using restriction enzymes is more important than the size of the DNA fragment and the location of the restriction site. Thus, *EcoR*1 was used to linearize the plasmid in the following study.

### Dynamic range of droplet digital PCR for quantifying plasmids

**Fig. 5:**
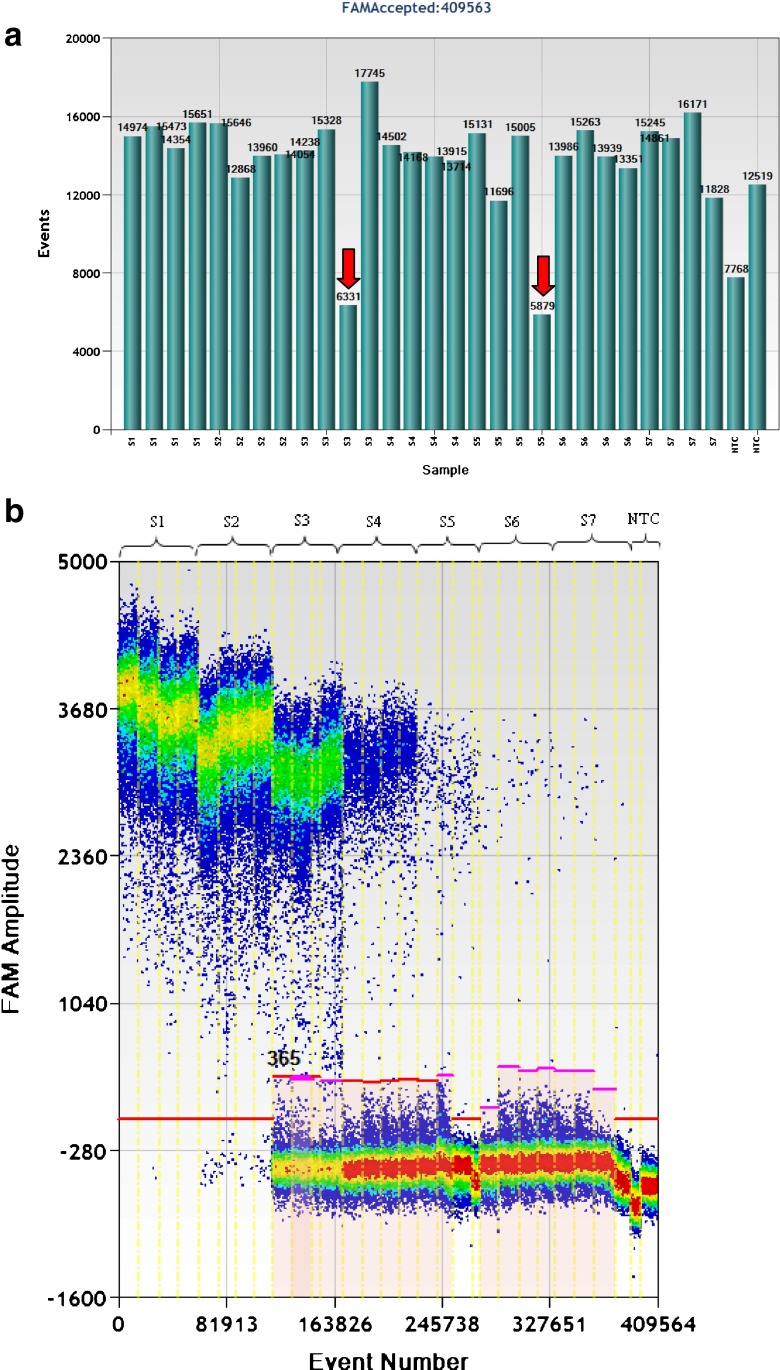

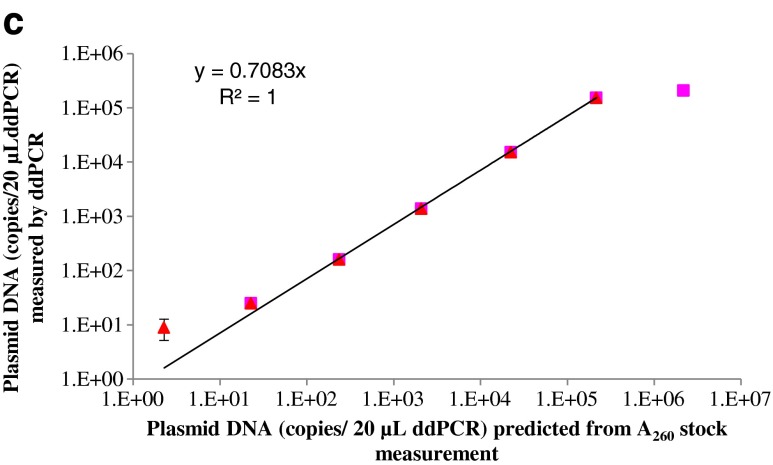
Evaluation of the droplet digital PCR method for quantification of linearized plasmid pNIM-003, digested by EcoR1 with AOB PCR assay

The ddPCR response over concentrations ranging from approximately 2.3–2,177,456 copies 20 μL^−1^ of ddPCR is shown in Fig. 5. The average number of accepted droplet events for all 30 reactions was 13,865, with a standard deviation of 2,449 (Fig. 5a), indicating successful droplet generation for all reactions. Because of the dead volume of the ddPCR reader, it is unable to estimate the true number of DNA targets if the number of droplets is too small when the DNA concentration is very low. Although there were two reactions with fewer than 10,000 droplets (see the arrows in Fig. 5a), this was not a problem because the concentration of DNA targets was high enough to generate a sufficient number of positive droplets. One-dimensional scatter plots of fluorescent droplet amplitudes for selected wells are shown in Fig. 5b. With restriction digestion, the positive droplets separated clearly from the negative droplets. However, the smear between positive and negative droplets still existed for the NECs (Fig. S2), suggesting unsuccessful amplification in initial cycles for these droplets. This is one factor causing underestimation of the true number of molecules by non-digestion treatment. Theoretically, there should be no negative droplets when the DNA concentration is high enough to saturate the generated droplets. In practice, this is true for the digested treatment (dilution S1 in Fig. 5a) but there are still negative droplets in the undigested plasmid (dilution S1 in Fig. S2), indicating unsuccessful single-molecule amplification of the non-linearized plasmid. This is another factor causing underestimation of the copy numbers of undigested plasmid.

The ddPCR response was linear over the dynamic range of 11–1081863 copies. The ddPCR response was linear over the dynamic range of 2.3–215 982 copies 20 μL^−1^ of ddPCR, covering five orders of magnitude. This is consistent with a previous study [19], in which the linearity range of ddPCR for quantifying lambda genomic DNA covered more than four orders of magnitude. The linearity regression of the ddPCR for quantifying pNIM-003 plasmid DNA is shown in Fig. 5c (*R* ^2^ = 1). In dilution S1, there were no negative droplets (Fig. 5c), indicating an over-saturation of the droplets. Thus, it is impossible to accurately estimate the copy number of the digested plasmid in dilution S1. The number of target DNA molecules of dilution S7 in ddPCR replicates is the most variable, because the copy number concentration of dilution S7 is the lowest (2.3 copies 20 μL^−1^). It is interesting to note that the data points for dilution S1 and dilution S7 are not a good fit to the linear curve in Fig. 5b. Thus, to achieve higher accuracy and better precision in ddPCR measurement, the DNA concentration should be within the optimum range.

Three subsamples of the plasmid diluted to the optimum concentration range were quantified using ddPCR. The measured copy number concentrations of the pNIM-003 of three subsamples are shown in Fig. S3. The concentrations of plasmid DNA in subsamples 1, 2, and 3 were determined to be 5.02 × 10^9^ copies μL^−1^, 5.10 × 10^9^ copies μL^−1^, and 5.03 × 10^9^ copies μL^−1^, respectively, and the RSDs were 3.33 %, 3.79 %, and 3.50 %, respectively. The average copy number concentration of the three subsamples was 5.05 × 10^9^ copies μL^−1^, with a relative standard deviation of 3.63 %. Those results were calculated based on the droplet volume of 0.846 nL measured in this study (Fig. S4, Electronic Supplementary Material); however, this will have been underestimated because of the inaccuracy of the droplet volume set in the software by the manufacturer (0.91 nL, personal communication from BioRad). It has been reported that the inaccuracy of the droplet volume could cause a bias in the ddPCR measurement. The droplet volume determined in this study is slightly different from that in an earlier report [19]. This is reasonable, because the 0.865 nL droplet size in the previous study was measured on a Beta-prototype instrument. The relative expanded uncertainty of the measurement result was 3.5 % (*k* = 2), evaluated by combining the uncertainty of the precision factor (*u*_*M*_), and variability of the droplet volume, the calibration of the microscope (*u*_*Vp*_), and the dilution factor (*u*_*D*_).

### Quantification by isotope dilution mass spectrometry (IDMS)

**Fig. 6:**
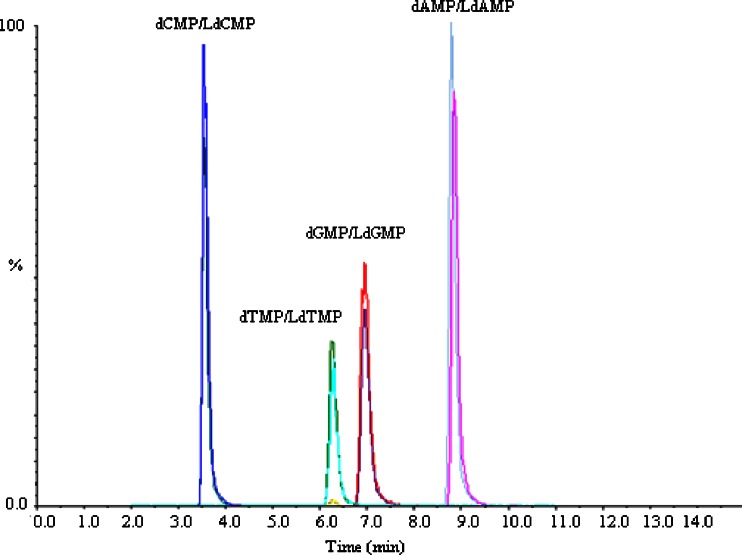
Total ion chromatography of the mixed-isotope labeled and unlabeled standard nucleotides obtained by liquid chromatography–tandem mass spectrometry

An independent method of IDMS was used to quantify plasmids of the same three subsamples. LC–IDMS is a well-established method, used for quantifying DNA in previous studies [20, 21]. The total ion chromatography (TIC) of the dNMPs and LdNMPs, shown in Fig. 6, reveals good separation of the four nucleotides by high performance liquid chromatography. For accurate IDMS analysis, it is essential that the ions of interest are free from mass-spectral interferences. Based on the multiple-reaction-monitoring (MRM) channels monitored for the dNMPs (Fig. S5), it is clear that there was no interference from any of the other dNMPs in the quantification of each dNMP.

**Fig. 7:**
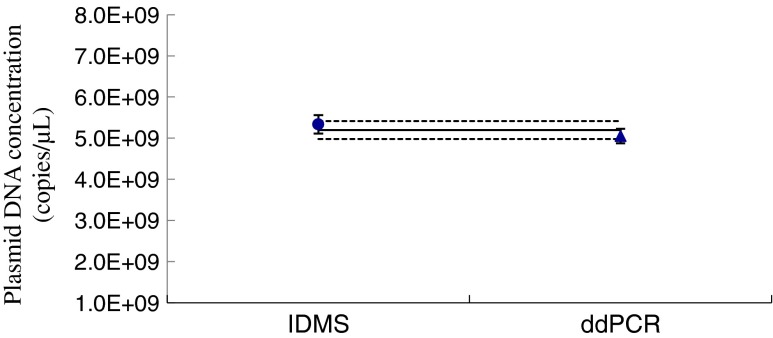
Measured plasmid pNIM-003 DNA concentrations, with the expanded uncertainty (*k* = 2) (copies μL^−1^), obtained by droplet digital PCR (ddPCR) and isotope dilution mass spectrometry (IDMS). The attributed concentration for the plasmid DNA stock (*continuous line*) with the expanded uncertainty (*dashed lines*) was calculated by averaging the two measurement results

The determined concentrations of plasmid DNA in three subsamples, calculated by Eq. 4, are shown in Fig. S3. The reported results were obtained from five repeat injections of each digested sample and calibration blend. The concentrations of plasmid DNA in subsamples 1, 2, and 3 were calculated as 5.35 × 10^9^ copies μL^−1^, 5.34 × 10^9^ copies μL^−1^, and 5.31 × 10^9^ copies μL^−1^, respectively, based on the measured dAMP concentration, whereas the RSDs were 3.11 %, 2.56 %, and 3.26 %, respectively. The uncertainty for each measurement was calculated by the method of uncertainty propagation described in JCGM [27]. The average concentration of the plasmid was 5.34 × 10^9^ copies μL^−1^, with an expanded uncertainty of 2.2 × 10^8^ copies μL^−1^ (*k* = 2).

### Determining the reference value of the plasmid reference material and its uncertainty

The average plasmid DNA concentration determined by IDMS and ddPCR is shown in Fig. 7. The result obtained by correction of the droplet size was 94.7 % of the IDMS value. The result from ddPCR measurement is approximately 5 % lower than the result from IDMS measurement, but it still can be overlapped within the expanded uncertainty, suggesting the bias for each measurement system has a minimal effect on the measurement of the plasmid reference material characterization. To assign the reference value for the plasmid pNIM-003, the precision of the two sets of data was checked, revealing equal precision of the two data sets from the two methods. Thus, the average value of the two measurement results was used as the reference value for the plasmid copy number concentration, which was (5.19 ± 0.22) × 10^9^ copies μL^−1^. Considering the contributions of homogeneity and stability to the uncertainty budget, the certified reference value with its expanded uncertainty (*k* = 2) is (5.19 × 0.41) × 10^9^ copies μL^−1^ (Table S3). The reference value of the plasmid reference material is more reliable and accurate when obtained by characterizing with two totally independent approaches. Therefore, the plasmid reference material with an accurate DNA copy number concentration is suitable for method validation and for quantifying ammonia oxidizer and denitrifier.

## Conclusion

DNA reference material with a certified value has a critical function in many analytical processes involving nucleic acids. In environmental microbiology, many studies focus on determining the distribution and abundance of ammonia oxidizers and denitrifiers in the natural environment by quantifying the functional gene, for example *amoA*, *nirS*, or *nosZ*. Comparability and reliability of analysis results can be achieved by using an accurate DNA reference material. Droplet digital PCR has applications in molecular genetic analysis, including DNA copy number measurement. One major advantage of ddPCR is that it is independent of DNA standards. However, for correct evaluation of data sets generated from ddPCR, it is crucial to consider sources of measurement bias. In this study, we revealed two major measurement biases when using ddPCR to quantify plasmid DNA. We demonstrated that restriction digestion of plasmid DNA into linearized plasmid DNA greatly increases amplification efficiency, and minimizes bias when measuring the true copy number. Compared with conformation of the plasmid, the size of the DNA fragment containing the target sequence and the location of the restriction site to the target sequence are not significant factors affecting plasmid quantification by ddPCR. Additionally, the droplet volume also contributes to the accuracy of the ddPCR measurement. In conclusion: consideration of the factors revealed in this study can improve the reliability and accuracy of ddPCR measurement. This gives ddPCR the potential to accurately quantify DNA reference material, which in turn underpins the quality and consistency of routine measurement. LC–IDMS, an independent DNA concentration measurement approach developed in our earlier study, was used to co-characterize the plasmid reference material with ddPCR. This will improve the accuracy and reliability of the plasmid reference material.

## Electronic supplementary material

Below is the link to the electronic supplementary material.

ESM 1 (PDF 1.11 mb)

## References

[1] Rønning SB, Vaïtilingom M, Berdal KG, Holst-Jensen A (2003) Event specific real-time quantitative. PCR for genetically modified Bt11 maize (Zea mays). Eur Food Res Technol 216:347

[2] Holst-Jensen A, Rønning SB, Løvseth A, Berdal KG (2003) PCR technology for screening and quantification of genetically modified organisms (GMOs). Anal Bioanal Chem 375:985 10.1007/s00216-003-1767-7 12733008

[3] Geets J, Cooma MD, Wittebolle L, Heylen K, Vanparys B, Vos PD, Verstraete W, Boon N (2007) Real-time PCR assay for the simultaneous quantification of nitrifying and denitrifying bacteria in activated sludge. Appl. Microbiol. Biotechnol. Appl Microbiol Biotechnol 75:211 10.1007/s00253-006-0805-8 17256118

[4] Liu X, Tiquia SM, Holguin G, Wu L, Nold SC, Devol AH, Luo K, Palumbo AV, Tiedje JM, Zhou J (2003) Molecular diversity of denitrifying genes in continental margin sediments within the oxygen-deficient zone off the Pacific coast of Mexico. Appl Environ Microbiol 69:3549 10.1128/AEM.69.6.3549-3560.2003 PMC161474 12788762

[5] Yoshida M, Ishii S, Otsuka S, Senoo K (2009) Temporal shifts in diversity and quantity of nirS and nirK in a rice paddy field soil. Soil Biol Biochem 41:2044

[6] Francis CA, Roberts KJ, Beman JM, Santoro AE, Oakley BB (2005) Ubiquity and diversity of ammonia-oxidizing archaea in water columns and sediments of the ocean. Proc Natl Acad Sci U S A 102:14683 10.1073/pnas.0506625102 PMC1253578 16186488

[7] Purkhold U, Pommerening-Roser A, Juretschko S, Schmid MC, Koops HP, Wagner M (2000) Phylogeny of all recognized species of ammonia oxidizers based on comparative 16S rRNA and amoA sequence analysis: implications for molecular diversity surveys. Appl Environ Microbiol 66:5368 10.1128/aem.66.12.5368-5382.2000 PMC92470 11097916

[8] Lashof DA, Ahuja DR (1990) Relative contributions of greenhouse gas emissions to global warming. Nature 344:529

[9] Wallenstein MD, Vilgalys RJ (2005) Quantitative analyses of nitrogen cycling genes in soils. Pedobiologia 49:665

[10] Gilbert Y, Bihan YL, Aubry G, Veillette M, Duchaine C, Lessard P (2008) Microbiological and molecular characterization of denitrification in biofilters treating pig manure. Biores Technol 99:4495 10.1016/j.biortech.2007.06.066 17935979

[11] Yan T, Fields MW, Wu L, Zu Y, Tiedje JM, Zhou J (2003) Molecular diversity and characterization of nitrite reductase gene fragments (nirK and nirS) from nitrate- and uranium-contaminated groundwater. Environ Microbiol 5:13 10.1046/j.1462-2920.2003.00393.x 12542709

[12] Dong L, Cordova-Kreylos AL, Yang J, Yuan H, Scow KM (2009) Humic acids buffer the effects of urea on soil ammonia oxidizers and potential nitrification. Soil Biol Biochem 41:1612 10.1016/j.soilbio.2009.04.023 PMC3260537 22267875

[13] Okano Y, Hristova KR, Leutenegger CM, Jackson LE, Denison RF, Gebreyesus B, Lebauer D, Scow KM (2004) Application of real-time PCR to study effects of ammonium on population size of ammonia-oxidizing bacteria in soil. Appl Environ Microbiol 70:1008 10.1128/AEM.70.2.1008-1016.2004 PMC348910 14766583

[14] Bhat S, Herrmann J, Armishaw P, Corbisier P, Emslie K (2009) Single molecule detection in nanofluidic digital array enables accurate measurement of DNA copy number. Anal Bioanal Chem 394:457 10.1007/s00216-009-2729-5 19288230

[15] Bhat S, Curach N, Mostyn T, Bains GS, Griffiths KR, Emslie KR (2010) Comparison of methods for accurate quantification of DNA mass concentration with traceability to the international system of units. Anal Chem 82:7185 10.1021/ac100845m 20690645

[16] Sykes PJ, Neoh SH, Brisco MJ, Hughes E, Condon J, Morley AA (1992) Quantitation of targets for PCR by use of limiting dilution. Biotech 13:444 1389177

[17] Vogelstein B, Kinzler K (1999) Digital PCR. Proc Natl Acad Sci U S A 96:9236 10.1073/pnas.96.16.9236 PMC17763 10430926

[18] Kalinina O, Lebedeva I, Brown J, Silver J (1997) Nanoliter scale PCR with TaqMan detection. Nucleic Acids Res 25:1999 10.1093/nar/25.10.1999 PMC146692 9115368

[19] Pinheiro LB, Coleman VA, Hindson CM, Herrmann J, Hindson BJ, Bhat S, Emslie KR (2012) Evaluation of a Droplet Digital Polymerase Chain Reaction Format for DNA Copy Number Quantification. Anal Chem 84:1003 10.1021/ac202578x PMC3260738 22122760

[20] Burke DG, Dong L, Bhat S, Forbes-Smith M, Fu S, Pinheiro L, Wang J, Emslie KR (2013) Digital polymerase chain reaction measured pUC19 marker as calibrant for HPLC measurement of DNA quantity. Anal Chem 85:1657 10.1021/ac302925f 23215355

[21] Dong L, Zang C, Wang J, Li L, Gao Y, Wu L, Li P (2012) Lambda genomic DNA quantification using ultrasonic treatment followed by liquid chromatography-isotope dilution mass spectrometry. Anal Bioanal Chem 402:2079 10.1007/s00216-011-5644-5 22218463

[22] De Leenheer AP, Lefevere MF, Lambert WE, Colinet ES (1985) Isotope-dilution mass spectrometry in clinical chemistry0 Adv Clin Chem 24:111 10.1016/s0065-2423(08)60272-3 3911749

[23] Hilpert K, Waidmann E (1988) Multi-element determination in environmental samples by mass spectrometric isotope dilution analysis using thermal ionization. Fresenius' Z Anal Chem 331:111

[24] Matsuoka T, Kuribara H, Akiyama H, Miura H, Goda Y, Kusakabe Y, Isshiki K, Toyoda M, Hino A (2001) A multiplex PCR method of detecting recombinant DNAs from five lines of genetically modified maize. Shokuhin Eiseigaku Zasshi 42:24 10.3358/shokueishi.42.24 11383153

[25] ISO guide 35:2006 Reference materials -- Reference materials - General and statistical principles for certification. General and statistical principles for certification

[26] Vazquez R, Steinberg M (1999) Evaluation of measurement data—guide to the expression of uncertainty in measurement. Biotechniques 26:91

[27] JCGM (2008) Evaluation of measurement data—guide to the expression of uncertainty in measurement Sèvres

