# Supplementary material for: Evaluation of droplet digital PCR for characterizing plasmid reference material used for quantifying ammonia oxidizers and denitrifiers
Source: Anal Bioanal Chem. 2014 Feb 4;406(6):1701–12. doi: 10.1007/s00216-013-7546-1 (PMC3936116; doi:10.1007/s00216-013-7546-1)
Supplement: Supplementary file 1 — (PDF 1.11 mb) [file 216_2013_7546_MOESM1_ESM.pdf]

Analytical and Bioanalytical Chemistry

Electronic Supplementary Material

Evaluation of droplet digital PCR on characterizing plasmid reference material used for quantifying ammonia oxidizers and denitrifiers

Lianhua Dong, Ying Meng, Jing Wang, Yingying Liu

1. Homogeneity study. For the homogeneity test, equation (1) to (9) was used for analysis the data. Where Q_1 is sum of squares between bottles, v_1 is degree of freedom between bottles, S_1^2 is mean sum of squares between bottles, Q_2 is sum of squares within bottles, is S_2^2 is mean sum of squares within bottles; v_2 is degree of freedom within bottles, n is number of measurements within bottles, m is the number of bottles, N is total number of measurements, F is the ratio of S_1^2 to S_2^2. To exam the homogeneity of the reference material, the F value was calculated by equation (1) to (9). If $F<Fa\ (v_1,v_2)$, the reference material is considered to be homogenous. Where $Fa\ (v_1,v_2)$ is the F value found in the F-distribution table with freedom of v_1 and v_2 at 95% confidence level. Additionally, if $F>1$, the $u_{bb,rel}$ the relative uncertainty of the inhomogenity, will be estimated by equation (8); if $F<1$, it will be estimated by equation (9).

$$Q_1 = \sum_{i=1}^{m} n_i(\overline{x_i} - \overline{\overline{x}})^2 \quad (1)$$

$$Q_2 = \sum_{i=1}^{m}\sum_{j=1}^{n_i}(x_{ij} - \overline{x_i})^2 \quad (2)$$

$$v_1 = m - 1 \quad (3)$$

$$v_2 = N - m \quad (4)$$

$$S_1^{\ 2} = \frac{Q_1}{v_1} \quad (5)$$

$$S_2^{\ 2} = \frac{Q_2}{v_2} \quad (6)$$

$$F = \frac{S_1^2}{S_2^2} \quad (7)$$

$$u_{bb} = \sqrt{\frac{s_1^2 - s_2^2}{n}} \quad (8)$$

$$u_{bb} = \sqrt{\frac{s_2^2}{n}} \bullet \sqrt[4]{\frac{2}{v_2}} \quad (9)$$

Table S1. Homogeneity analysis of plasmid pNIM-003 reference material by droplet digital PCR (m = 15, n = 3)

Bottles (m)	Concentration of subsamples (n) (copies/µL)		
	1	2	3
1	4.96E+09	5.04E+09	5.04E+09
2	4.93E+09	5.14E+09	5.20E+09
3	5.12E+09	5.05E+09	5.09E+09
4	5.48E+09	5.08E+09	5.67E+09
5	5.23E+09	5.20E+09	4.94E+09
6	5.08E+09	5.53E+09	5.17E+09
7	5.10E+09	5.13E+09	5.13E+09
8	5.10E+09	4.93E+09	5.10E+09
9	5.01E+09	5.13E+09	5.05E+09
10	5.28E+09	5.26E+09	5.15E+09
11	5.12E+09	5.75E+09	4.84E+09
12	5.49E+09	5.15E+09	4.98E+09
13	4.96E+09	4.84E+09	4.67E+09
14	5.46E+09	5.17E+09	4.88E+09
15	5.06E+09	5.14E+09	4.97E+09
Q_1		9.73E+17	
v_1		14	
S_1^2		6.95E+16	
Q_2		1.58E+18	
v_2		30	
S_2^2		5.26E+16	
F		1.32	
$Fa(v_1,v_2)$		2.04	
$u_{bb,rel}$		1.5%	

2. Stability study. For the examination of the long term stability of the plasmid pNIM-003 reference material, the data of plasmid concentration measured at different time points was used to generate a stability regression line with a regression equation of Y= $b1$x+$b0$. Where $b1$ is the slope of the regression line and $b0$ is the intercept of the line. $s_{(b1)}$, representing for the standard deviation of the slope, is estimated by equations (10) and (11). If $|b1| < s(b1) * t_{0.95,4}$, the statistic analysis reveals that there is no significant trend at 95 % confidence level for the stability regression line generated as described above. Where $t_{0.95,4}$ is the t value listed in the T-distribution table with the freedom of 4 at the confidence level of 95%. Given a shelf life of t, 12 months, $u_{S,rel}$, the relative uncertainty of instability, is estimated by $s(b1)$ multiply t and then divided by the measured mean value.

$$s(b1) = \frac{s}{\sqrt{\sum_{i=1}^{n}(x_i - \bar{x})^2}} \qquad (10)$$

$$s = \sqrt{\frac{\sum_{i=1}^{n}(y_i - b0 - b1x_i)^2}{n-2}} \qquad (11)$$

Table S2. Analysis of long term stability of plasmid reference material

Storage time (months)	Concentration (copies/µL)
0	5.09E+09
1	5.15E+09
2	5.12E+09
4	5.14E+09
7	5.09E+09
12	5.14E+09
Parameters	value
$\lvert b1 \rvert$	1.00E+06
$s_{(b1)}*t_{0.95,4}$	3.98E+07
$s_{(b1)}*t$	1.75E+08
Conclusion	Stable
$u_{S,rel}$	0.03

3. Droplet volume measurement. Droplets used for optical imaging to determine droplet volume were prepared using the same reagents as used for ddPCR estimation of the plasmid DNA concentration. Two wells were randomly selected from each of four different droplet generator cartridges. Twenty droplets were measured from each channel providing a total measurement of 160 droplets. The droplets were pipeting into a microscopy chambers coated with a coverslip and images were captured with a 20× objective of a Zeiss Observer Z1 inverted fluorescence microscope using an AxioCam HRm digital CCD camera. Droplet images were recorded under uniform illumination in a bright field with an illumination source of a 120 W halogen lamp. The Axiovision software version 4.6 was employed to analyze the digital images.

The digital image was analyzed by ImageJ v1.34s and the process for determining the equivalent circular diameter and the equivalent spherical volume of a sphere referred the previous report by Pinheiro 2012. Briefly, the droplet image was converted to a bit depth of 8 bit and the edge of the droplet was identified with function of "find edge" in the ImageJ software. Then, the edge of the droplet was defined by thresholding the inverted image. The major and minor axises of an elliptical fit to the droplet outline were determined on the binary image of the droplet, and then used to calculate the area in $(\text{pixels})^2$ of an ellipse of equivalent dimensions. The equivalent circular diameter was converted to length unit. Finally, the equivalent spherical volume of a sphere can be calculated based on the equivalent circular diameter.

Table S3. Uncertainty buddget of the plasmid pNIM-003 reference material

Uncertainty source	$u_{m,rel}$	$u_{S,rel}$	$u_{bb,rel}$	$u_{c,rel}$	U_{rel} (k=2)	U (k=2)
Uncertainty	2.1%	3.0	1.5%	4.0%	8.0%	0.41×10^9

$u_{m,rel}$, relative uncertainty of the method used for determining the reference value;

$u_{S,rel}$, relative uncertainty of the instability;

$u_{bb,rel}$, relative uncertainty of the inhomogeneity;

$u_{c,rel}$, combined relative uncertainty;

$U_{rel,}$ relative expanded uncertainty;

U, expanded uncertainty;

k, the coverage factor

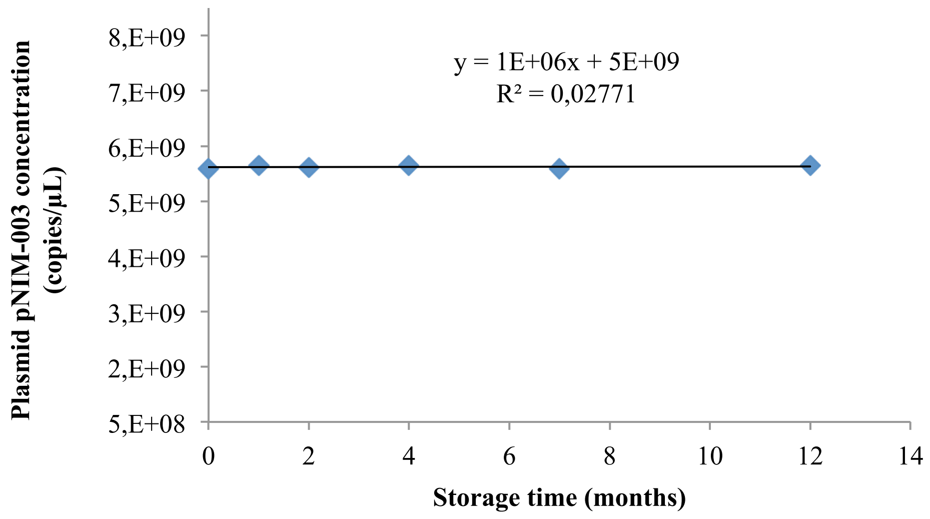

Fig S1. The stability regression line

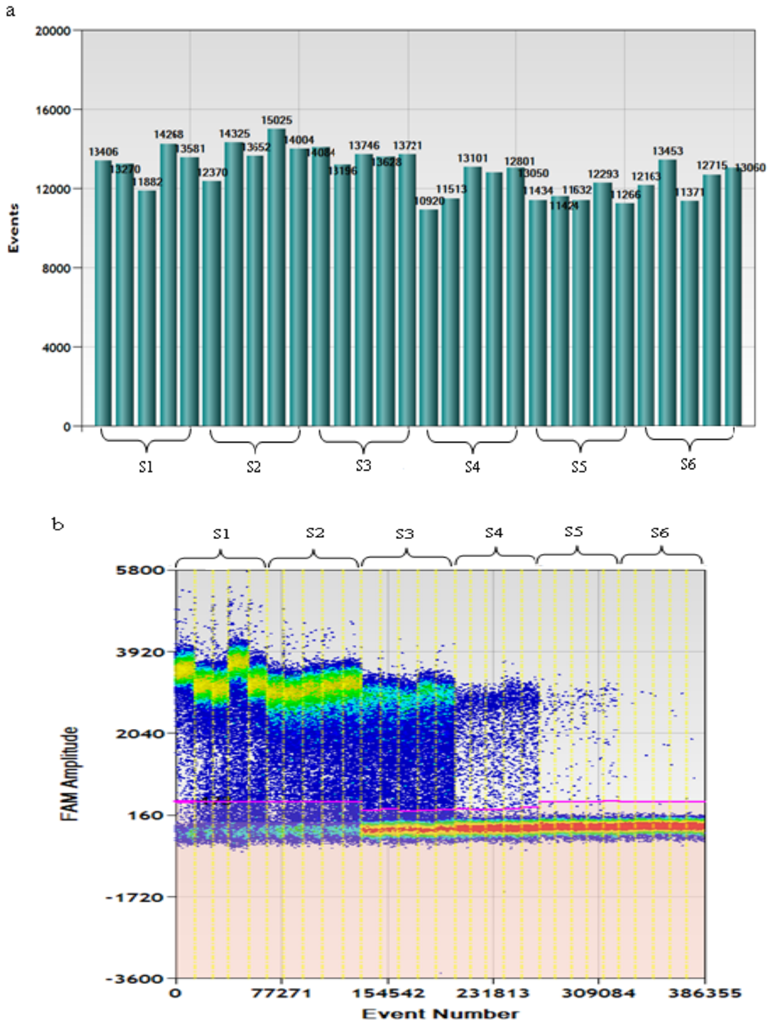

Fig. S2. Evaluation of the droplet digital PCR method for quantification of undigested plasmid pNIM-003. a, The number of droplet events counted for selected wells/samples; b, One-dimensional scatter plots for selected wells/samples fluorescent droplet amplitudes

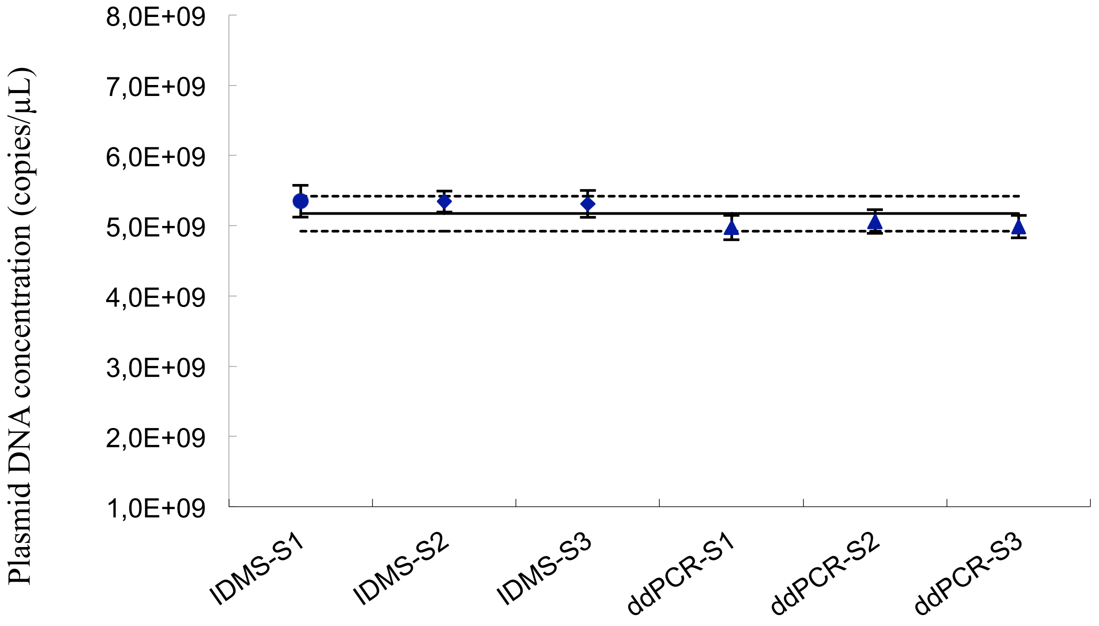

Fig. S3. Measured plasmid pNIM-003 concentration (copies/μL) for the three subsamples showing their expended uncertainty (*k*=2) by isotope dilution mass spectrometry (IDMS) and droplet digital PCR (ddPCR). The solid line represents the averaged number of all values, the dash line represents the expended uncertainty of the averaged number (*k*=2)

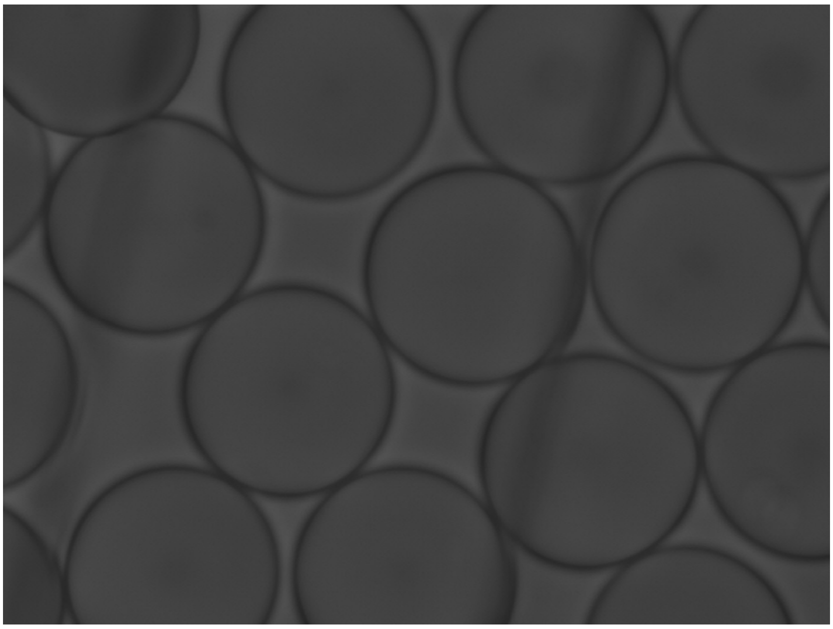

Fig. S4. The image of the droplets scanned by Zeiss Aixo Observer.Z1 microscope with 20× field lens. The droplets were pipette into a blood counting chamber covered with a microscopic glass to form a single layer

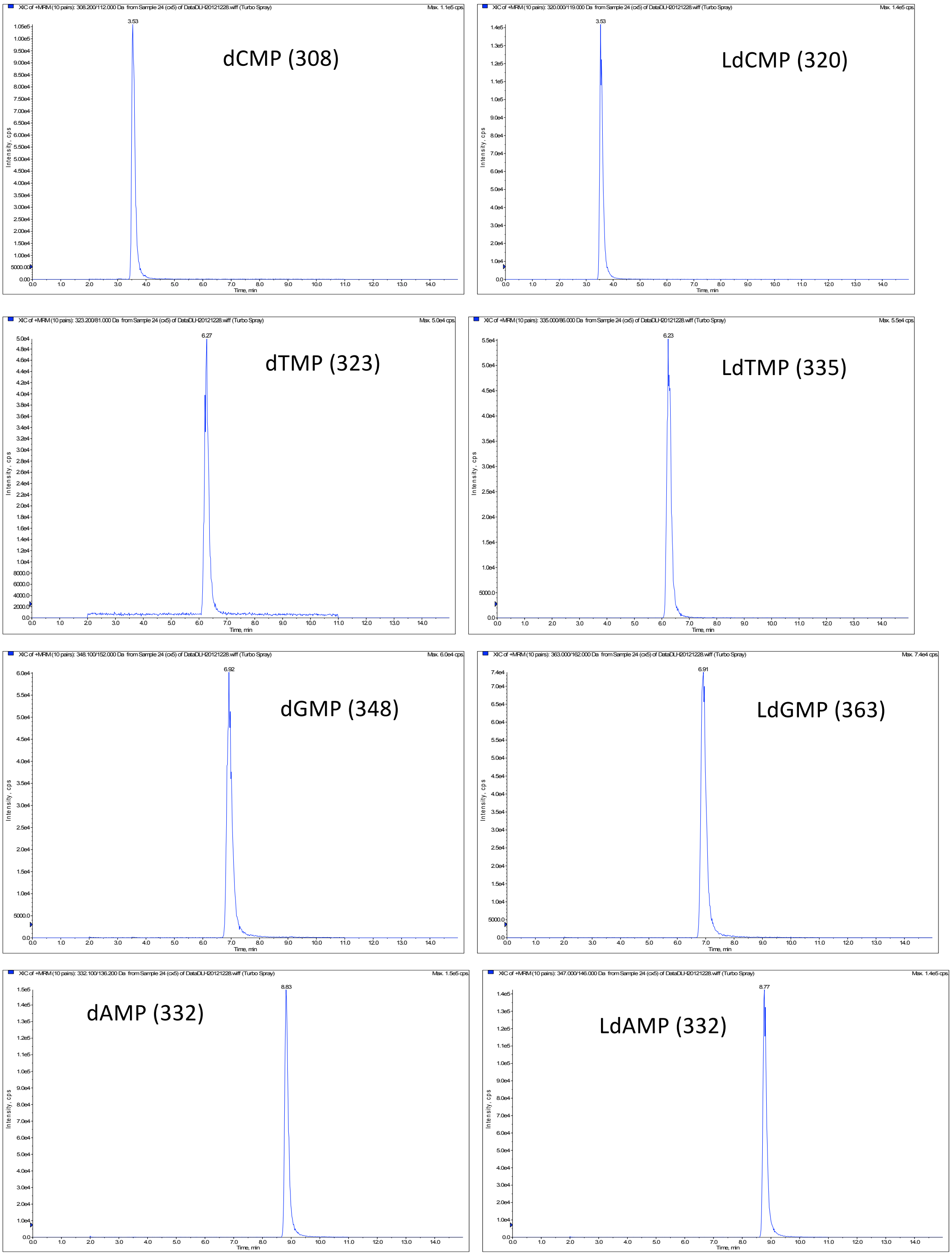

Fig. S5. The extracted ion chromatograph of multiply reaction monitoring
